# Formulation and validation of mathematical model for co-sputtering conditions to attain stoichiometric CZTS films: Expedience of using all metal sulfide targets

**DOI:** 10.1016/j.heliyon.2025.e41758

**Published:** 2025-01-09

**Authors:** Munira Sultana, Afrina Sharmin, Md Rashed Alam, Shahran Ahmed, Md Aftab Ali Shaikh, M.S. Bashar

**Affiliations:** Bangladesh Council of Scientific and Industrial Research (BCSIR), Bangladesh

**Keywords:** Co-sputtering deposition, CZTS thin films, Deposition pressure modulation, Material properties correlation, Sulfurization effect in CZTS fabrication

## Abstract

A soda lime glass substrate is used for fabricating Cu_2_ZnSnS_4_ (CZTS) thin films using copper (II) sulfide (CuS), zinc sulfide (ZnS), and tin sulfide (SnS) targets using an advanced co-sputtering deposition process. Following that, the films are annealed at 470 °C without sulfur (S). An algorithm based on the deposition rate of the previously specified targets set the co-sputtering condition, which maintains a deposition pressure of 5, 10, 15, and 20 mTorr. When studied at different deposition pressures, carrier concentration, resistivity, band gap, and crystallite size show significant correlations. The systematic variation of deposition pressure (5–20 mTorr) shows a constant increase in crystallite size. Resistivity decreases with pressure, but the band gap rises. The film has the highest resistivity at 5 mTorr Argon (Ar) deposition pressure and the highest carrier concentration at 10 mTorr. The EDX study shows that annealed films have a good stoichiometric ratio without sulfurization. Stoichiometry control, energy economy, and process simplicity improve when sulfurization is skipped for CZTS manufacturing. The findings explain correlations according to the intended use, thus experimentalists in this field will be interested in them.

## Graphical abstract

**Figure:**
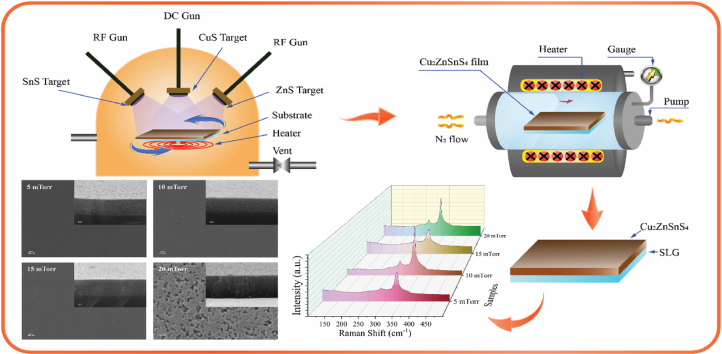

## 1 Introduction

Growing population and higher earnings are predicted to cause global energy consumption to increase to 660 quadrillion Btu in 2050. To address this issue, solar power might be a dependable solution. The c-Si-based solar panels have maximum efficiency in the present market with a high production cost. At the same time, thin-film solar cells have made it possible to produce solar electricity at a cheap cost with high conversion efficiency. Among them, CdTe, CIGS, and a-Si-based solar cells have an uprise in commercial production. However, due to the unavailability of In, Ga, and toxicity of Cd, these types of solar panels are becoming unpopular regarding the cost value or cost safety issues. The components of Kesterite CZTS are abundant and non-toxic. The highest efficiency of CZTS was obtained 12.6 % in 2014 [1]. While CZTS solar cells have a 32.2 % theoretical efficiency [2], it is not achieved because of various significant aspects, including how well the absorber layer performs, contact interface, and heterojunction. The CZTS solar cells efficiency is drastically reduced by severe interface recombination brought on by secondary phases, high concentrations of defects, and “cliff”-like conformation [3,4]. Researchers are trying to overcome these issues and take the benefit of low-cost, earth-abundant CZTS. According to Lu et al., post-heat treatment may lengthen the time that Cd diffuses as well as encourage the dispersion of Cu and Zn toward the buffer layer. That guarantees heterojunction CZTS/CdS of the highest quality with increased solar cell efficiency [5]. The Cd/In dual buffer layer was studied by Campbell et al. to enhance carrier collection, extraction, and J_sc_ of CZTSSe devices [6]. Band alignment type of CZTS devices is also responsible for lower power conversion efficiency (PCE). Li et al. altered the band alignment of the CZTS device by grain boundary oxidation and could enhance 30 % PCE [7]. Many studies show that alkali metals such as Li [8,9], Na [10,11], K [12], and Pd [13] can passivate defects. Rehan et al. studied different Na incorporation routes. They suggest Na Bifacial treatment method may be the best option to raise the efficiency of solar cells [14]. Zhao et al. introduced UV-ozone gas during the fabrication of CZTS films to remove organic compounds and promote larger grain growth to improve device performance [15].

Because it is a volatile substance, sulfur (S) is prone to evaporating during the heat treatment of the sputter-deposited thin films. Sulfurization is a high-temperature process to incorporate S into the annealed thin films to attain the standard stoichiometric ratio. It is a vital step in order to create crystalline CZTS thin films with the desired stoichiometric ratio [16,17]. However, it is a high-temperature and health-hazardous method. Moreover, toxic H_2_S was often used as sulfur source material [[18], [19], [20]].

Based on the provided findings and existing knowledge, the research presents an approach to fabricating Cu_2_ZnSnS_4_ (CZTS) thin films using a co-sputtering method with optimized deposition process parameters. Sputter deposition allows for better control over the deposition process to enhance the reproducibility of thin films, which is crucial for the manufacturing of solar cells. In addition, co-sputter deposition using CuS, ZnS, and SnS targets are employed for contributions to existing knowledge in the field of CZTS thin film solar cell technology. This work also focuses on the influence of deposition pressure on film properties i.e. optimization of crystallite size, enhancing morphology, band gap engineering, understanding resistivity, and carrier concentration. A sulfur source-free annealing process at 470 °C is explored for simplifying fabrication techniques and reducing processing steps, potentially leading to cost-effective and scalable production methods. Consequently, there is abundant sulfur content in co-sputter deposited CZTS and the heat-treated films were able to achieve a satisfactory stoichiometric ratio. Indeed, the noteworthy outcomes from the optimized deposition method and annealing process in the absence of a specific sulfur source are intriguing.

## 2 Method

### 2.1 Materials

**Fig. 1:**
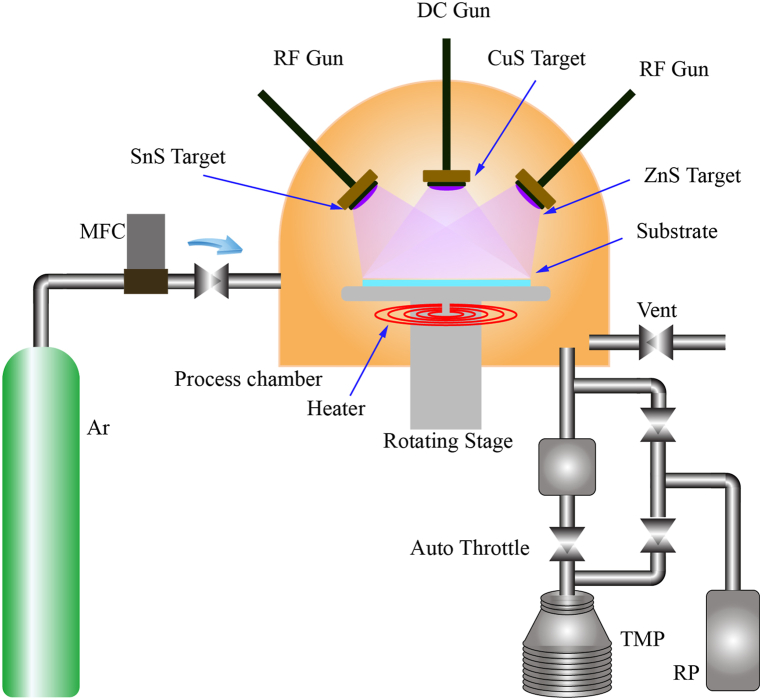
Schematic representation of the magnetron sputtering system.

The deposition of Cu_2_ZnSnS_4_ thin films was carried out utilizing the NSC-4000 top-down magnetron sputtering equipment, which was built by Nano-Master Inc, USA. The schematic diagram of the system is depicted in Fig. 1. Three sputter targets—CuS (Super Conductor Materials, Inc., 99.99 % purity), ZnS (Kurt J. Lesker Company, 99.99 % purity), and SnS (Angstrom Sciences, Inc., 99.99 % purity)—each with a thickness of 2.5 inches and a diameter of 2 inches, were mounted vertically with their surfaces oriented parallel to the substrate holder's spinning stage. The power sources that were associated with these objectives included RF1 (with a maximum power of 300 W and a frequency of 13.56 MHz), RF2 (with a maximum power of 300 W and a frequency of 12.56 MHz), and DC (with a maximum power of 600 W).

The experimental configuration included soda lime glass (SLG) substrates of dimensions 30 × 30 mm and a thickness of 1.5 mm. The substrates were situated at a distance of approximately 10 cm beneath the objectives. The chamber had a substrate holder that was affixed to a spinning stage, which had the ability to achieve a maximum speed of 20 revolutions per minute. The spinning stage was connected to a resistive heater with the ability to reach temperatures of up to 300 °C.

Prior to the deposition process, the SLG substrates were subjected to a cleaning protocol that involved a sequential treatment with three solutions, namely methanol, acetone, and methanol. This cleaning operation was carried out using an ultrasonic bath, with each solution being applied for 10 min. Subsequently, SLG substrates underwent a rinsing process utilizing deionized (DI) water, followed by a drying procedure employing a dry air blower.

### 2.2 Optimization of sputtering condition for individual targets

The co-sputtering condition was selected by evaluating the individual target's (CuS, ZnS and SnS) deposition properties. In order to enhance the deposition process, it was ensured that the base pressure within the chamber remained consistently below 5.5**×**10^−6^ Torr. CuS, ZnS, and SnS thin films were deposited at various DC/RF powers between 20 and 50 W. Thin film thickness measurements were used to determine the deposition rate. Calculated deposition rates are listed in Table 1 for different DC/RF powers. For all CuS, ZnS, and SnS thin films, the substrate temperature was set to 180 °C during sputter deposition, with the deposition pressure maintained at 5 mTorr.

**Table 1:** Rate of deposition (nm/min) of individual targets.

Power	CuS (DC)	SnS (RF)	ZnS (RF)
20	2.31	3.46	0.79
30	3.70	6.72	1.42
40	5.53	9.98	2.09
50	6.47	12.00	2.88

**Fig. 2:**
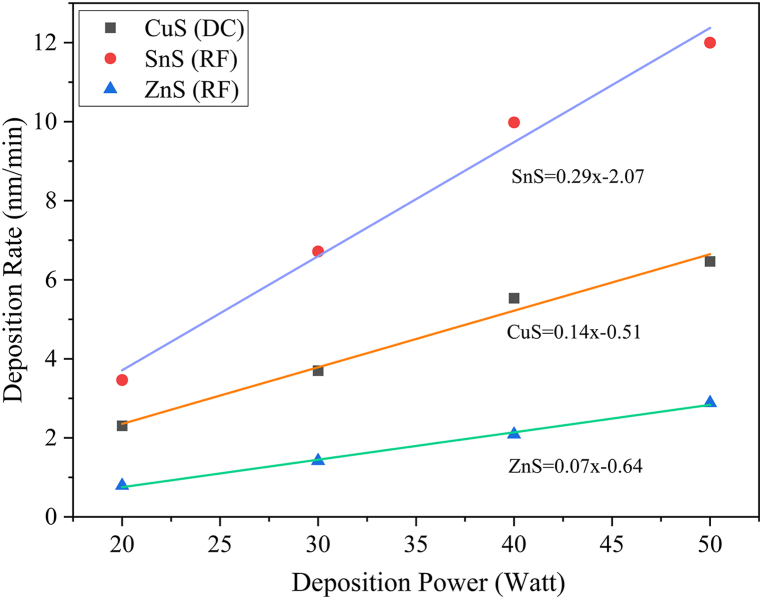
Deposition rate for sputtered film using SnS, CuS and ZnS targets with varying deposition power.

Fig. 2 demonstrates the trendline for individual targets. The ZnS thin films deposition rate increases linearly from 0.79 to 2.88 nm/min when the power was varied from 20 to 50 W.

Whereas 3.46 nm of SnS thin films are deposited per minute at 20 W, this rate grows linearly to 12 nm every minute. The film deposition rate for the CuS target was initially 2.31 nm/min, which increases linearly to 6.47 nm/min.

Linear correlation coefficient (R) for CuS, ZnS, and SnS are 0.9863, 0.9969, and 0.9890 respectively, which relates the deposition power to the deposition rate. The yield equations (1), (2), (3) concerning unknown deposition power as x for CuS, ZnS and SnS targets, are as follows,

(1) CuS=0.1431x−0.5073

(2) ZnS=0.0695x−0.6394

(3) SnS=0.2888x−2.0659

The average atomic percentage of individual elements from the EDS report of sputter-deposited thin films using individual targets at different powers is listed in Table 2.

**Table 2:** Elemental analysis of thin film deposited from individual target.

Target	Element	Deposit (%At)
CuS	Cu	71.00
	S	29.00
ZnS	Zn	32.00
	S	68.00
SnS	Sn	46.93
	S	53.07

The sputter-deposited films have different elemental compositions other than the target materials. The best CZTS device performance was obtained using Zn-enriched and Cu-deficient films, as has been documented in the literature. For that, Cu to Zn and Sn will have a 0.95 ratio and Zn to Sn will be around a 1.10 ratio [21].

Assuming the deposition rate (DR) of SnS target, ${DR}_{SnS}=2.00\;nm/\min$, deposition rate of CuS target (DR_CuS_) and deposition rate of ZnS target (DR_ZnS_) were calculated using the following equations:

(4) $${DR}_{CuS}=\frac{\frac{Cu}{Zn+Sn}*{\%At}_{Sn}*\left(1+\frac{Zn}{Sn}\right)*{DR}_{SnS}}{{\%At}_{Cu}}\;nm/\min$$

(5) $${DR}_{ZnS}=\frac{{DR}_{SnS}*{\%At}_{Sn}*\frac{Zn}{Sn}}{{\%At}_{Zn}}\;nm/\min$$

%At_Sn_, %At_Cu_, and %At_Zn_ are the atomic percentages of Sn, Cu, and Zn respectively in sputter-deposited films using individual targets.

The power for each target can be calculated using equations (6), (7), (8) which relate the goal composition, yield equation, and elemental analysis to reach the predicted ratio for depositing CZTS thin film, as below,

(6) $$\text{The}\;\text{power}\;\text{for}\;\text{the}\;\text{CuS}\;\text{target}=\frac{{\text{DR}}_{\text{CuS}}‐C_{\text{CuS}}}{m_{\text{CuS}}}\;W$$

(7) $$\text{The}\;\text{power}\;\text{for}\;\text{the}\;\text{ZnS}\;\text{target}=\frac{{\text{DR}}_{\text{ZnS}}‐C_{\text{ZnS}}}{m_{\text{ZnS}}}\;W$$

(8) $$\text{The}\;\text{power}\;\text{for}\;\text{the}\;\text{SnS}\;\text{target}=\frac{{\text{DR}}_{\text{SnS}}‐C_{\text{SnS}}}{m_{\text{SnS}}}\;W$$

DR_CuS,_ DR_ZnS,_ and DR_SnS_ are the calculated deposition rates to achieve the goal composition for CuS, ZnS, and SnS thin films respectively (Table 3). C_CuS,_ C_ZnS,_ and C_SnS_ are the y-intercept points of the trendlines for CuS, ZnS, and SnS targets in Fig. 2 m_CuS,_ m_ZnS,_ and m_SnS_ are the slope of the deposition rate versus the deposition power curve in Fig. 2. Finally, the parameters for the co-deposition of CuS, ZnS, and SnS are calculated and listed in Table 4.

**Table 3:** Deposition rate from equations (4), (5) to achieve the goal composition of CZTS.

Deposition rate (nm/min)	
CuS	2.64
ZnS	3.23
SnS	2.00

**Table 4:** Calculated co-deposition power for individual target.

CuS (DC power)	22 W
ZnS (RF power)	56 W
SnS (RF power)	14 W

### 2.3 CZTS thin film fabrication

One DC target and two RF two-inch confocal targets were used in a magnetron sputtering setup to deposit the CZTS thin films. Ultrasonically cleaned SLG were used as substrates for fabricating the CZTS absorber layer for solar cells. Just before deposition, the cleaned substrates were plasma treated for 2 min to remove any residual contaminants. CuS, ZnS, and SnS targets were respectively connected to DC (22 W), 1RF (56 W) and 2RF (14 W) power sources. Onto pre-cleared soda lime glass substrates, sputter deposited CZTS thin films were applied. Targets and substrates were separated by a predetermined 10 cm spacing. Pure Argon (Ar) gas was injected at a chamber pressure of $5.5\times 10^{-6}$ Torr. The pressure within the chamber rose when the Ar gas flow started, and the targets sputtered after attaining a stable chamber pressure with steady gas flow. To examine how sputtering pressure affects the optical absorption coefficient, band gap, chemical composition, crystallinity, and electrical characteristics of CZTS films, the operating pressure was adjusted within a range of 5–20 mTorr by regulating the flow of Ar gas. To indicate the matching deposition pressures of 5, 10, 15, and 20 mTorr, the CZTS films were given the labels C5, C10, C15, and C20. Furthermore, the substrate's temperature and rotation rate were preset at 180 °C and 20 rpm, respectively. The sputtering period was somewhere between 60 and 134 min in order to keep the CZTS thin film thickness fixed at around 850 nm for various operating pressures and make the film characteristics comparable for various analyses. Sputtered-deposited CZTS samples were annealed for 30 min at 470 °C in a 450 Torr nitrogen (N₂) environment. No sulfur powder or hydrogen sulfide gas was introduced during annealing. This low-temperature, high-pressure annealing configuration was designed to minimize sulfur loss while achieving good stoichiometry.

### 2.4 Characterization

A surface profilometer (DektaXT-A; Bruker Corporation, Swedesboro, NJ, USA) measured the film thickness using a diamond stylus of 2 μm diameter. The elemental and morphological properties were observed using a scanning electron microscope with energy dispersive analysis of X-ray (SEM, EDX) (EVO 18; ZEISS, UK). Using Cu Kα radiation (λ = 0.15406 nm) at 35.5 kV and 28 mA, an X-ray diffractometer (XRD) (EMMA; GBC Scientific Equipment, Braeside, Australia) was used to analyze the crystalline phase of the films. An ultraviolet–visible–near-infrared (UV–Vis–NIR) spectrometer (UH-4150; Hitachi High Technologies Corporation, Tokyo, Japan) was used to assess the optical characteristics of the films. Atomic force microscopy (AFM) was used to analyze the films' surface topology and roughness (C3000, FlexAFM; Nanosurf AG, Liestal, Switzerland). A Raman spectrophotometer (LabRAM HR Evolution, Horiba Scientific, Japan) with a 532 nm laser was used for all the Raman scattering studies. In order to excite the samples, the laser beam was focused using a 100 × objective lens onto a point with a diameter of 5 μm. The Raman spectra were acquired 10 times with an integration duration of 5 s. Electrical properties were measured with hall effect measurement unit (HMS-3300, Ecopia Corporation, South Koria).

## 3 Results and discussion

### 3.1 Deposition rate

**Fig. 3:**
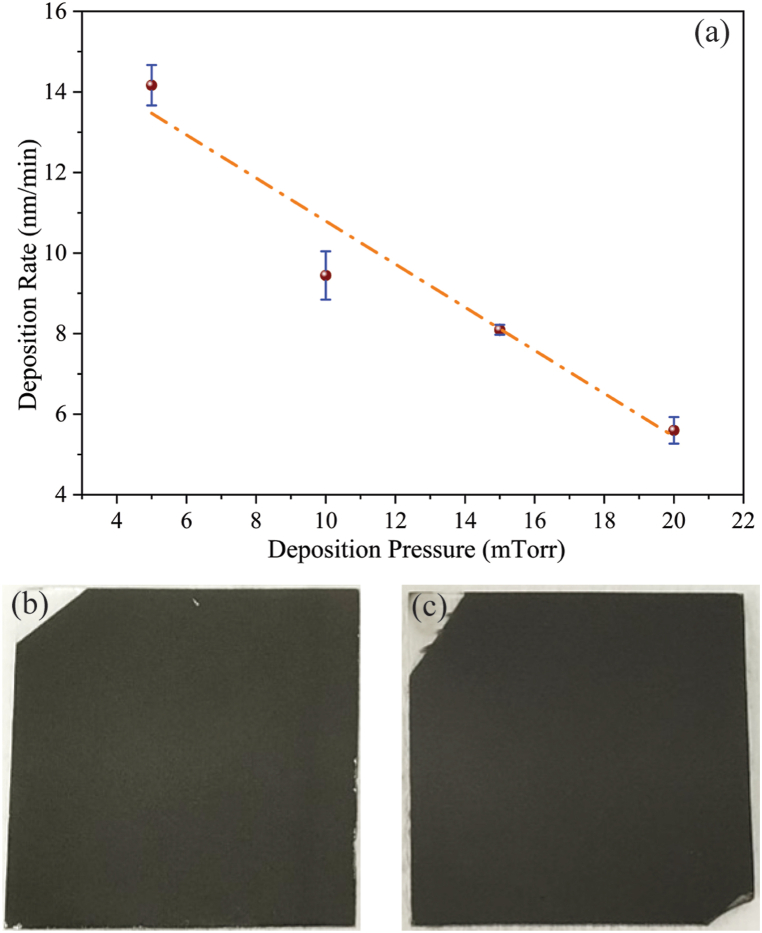
(a) CZTS film deposition rate dependence on sputtering pressure, (b) CZTS sputter deposited film on a glass substrate, and (c) CZTS thin film after annealing.

The deposited films had excellent substrate adhesion and were physically stable on the soda lime glass substrates. After annealing no break or peeling of the films was seen as shown in Fig. 3(a) illustrating how sputtering pressure affects the pace at which CZTS films are deposited.

Due to the increased frequency of sputtered molecules colliding with gas molecules and thermalizing the film-forming particles, the deposition rate reduced with growing pressure [22]. As a result, the atoms or molecules capacity to diffuse lessens, which lowers the rate of deposition. In order to maintain an average CZTS thin film thickness of 850 nm, the deposition period was modified. Thus, the comparability of all the analysis outcomes is guaranteed.

Fig. 3(b) shows a sputter-deposited CZTS thin film on a glass substrate, exhibiting a uniform surface coverage. The deposition process ensures a smooth and adherent film layer, which forms a foundation for further processing. After annealing, as shown in Fig. 3(c), the CZTS thin film demonstrates improved crystallinity and structural integrity. The annealing process enhances the material properties, which are crucial for optimizing the film's performance in photovoltaic applications.

### 3.2 Elemental analysis

From the EDX measurement reported in Table 5, the compositional analyses of the fabricated thin films were obtained. Any variation from the optimal stoichiometry ratio causes secondary phases and defects to appear. Table 5 makes clear that when the deposition pressure increases, the Cu concentration of the sputtered CZTS films rises. Compared to the other two elements in CZTS, Sn and S have lower melting temperatures and are more volatile. However, the atomic percentage of S and metals in the sputtered samples indicates that the sputtered samples contained an adequate quantity of sulfur, which facilitated the formation of the kesterite phase during annealing. Although Sn and S are volatile elements with lower melting points, the annealed samples retained sufficient sulfur to achieve good stoichiometry. For instance, sample C10 exhibited a stoichiometric ratio close to 2:1:1:4, which is optimal for CZTS thin films used in solar cell applications. Additionally, the reduced annealing temperature avoids excessive volatility of sulfur and tin, which can disrupt the composition. These findings align with prior research (e.g., Rakhshani and Thomas [21]) that suggest slightly Zn-enriched and Cu-deficient compositions improve the efficiency of CZTS thin films in solar cell applications. The C5 has an excess amount of S, whereas C20 has Sn content after annealing. The growing component volatility loss or compounds formed at high annealing temperatures, having a considerable effect on the composition's ultimate form is believed to be the cause of this undesired phenomenon.

**Table 5:** Compositional analysis of the CZTS thin films.

Sample	Sputtered	Zn/Sn	S/Metal	Stoichiometry	Annealed	Zn/Sn	S/Metal	Stoichiometry
	Cu/(Zn + Sn)				Cu/(Zn + Sn)			
C5	0.76	0.80	1.67	1.4/0.8/1/5.4	0.85	0.97	1.28	1.7/1/1/4.7
C10	0.92	1.09	0.99	2/1.1/1/4	1.10	0.91	0.98	2.1/1/1/4
C15	1.08	1.09	0.95	2.3/1.1/1/4.2	0.89	1.22	1.05	2/1.3/1/4.4
C20	1.54	1.29	1.03	3.6/1.3/1/6	1.38	0.23	0.62	1.7/0.3/1/1.9

### 3.3 Structural studies

**Fig. 4:**
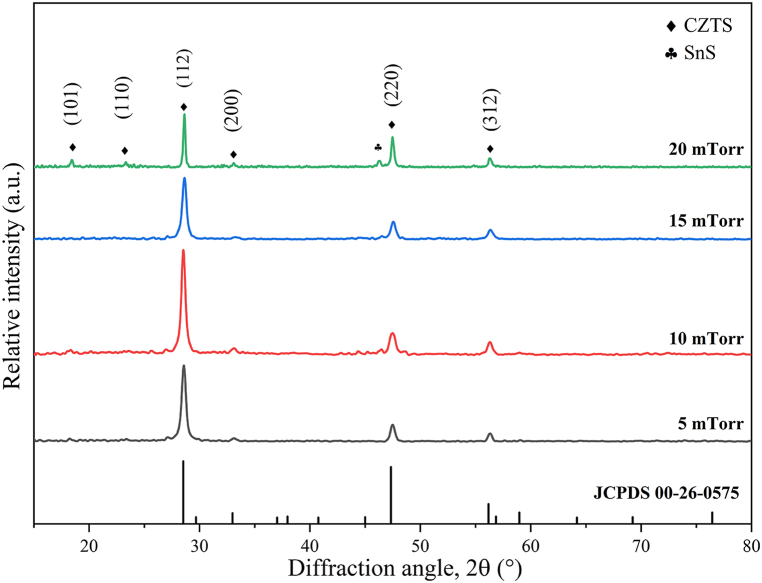
XRD patterns for the CZTS films deposited at different sputter pressures.

Through annealing, kesterite-like polycrystalline CZTS thin films were produced. According to the card JCPDS 26–0575, as can be observed in Fig. 4, reflections are allocated to the (101), (110), (112), (200), (220), and (312) planes with the (112) plane having favored orientation in all X-ray diffraction patterns.

No matter the method of deposition, secondary phase development in quaternary compounds like CZTS is difficult to avoid. From Fig. 4, it is evident that the films also include the SnS (JCPDS Card no. 03-065-3766) phase in addition to the CZTS kesterite phase [23], which is prominent for the films deposited at 20 mTorr. EDX report from Table 6 also confirms the excess amount of Sn content in the sputtered CZTS film at 20 mTorr, which may result in the formation of a SnS secondary phase in the annealed films.

**Table 6:** Structural parameters of the CZTS thin films.

Sample	FWHM of (112) plane *β* (°)	Crystallite size *D* (Å)	Dislocation density *δ* (10^−3^nm^−2^)	Micro-strain Ε (10^−2^)	FWHM of Raman peak at 335 cm^−1^	Lattice parameters(nm) (Rietveld-refined structure)		
						a	c	c/a
C5	0.417	205	2.37	0.407	14.58	5.404	10.888	2.01
C10	0.402	213	2.20	0.397	10.89	5.417	10.848	2.00
C15	0.409	209	2.28	0.400	19.61	5.420	10.901	2.01
C20	0.196	437	5.23	0.192	11.74	5.471	10.931	1.99

To calculate the average crystallite sizes (D) of the films, the Scherrer formula is utilized [24].

(9) $$D_{hkl}=\frac{0.9}{\cos\;\theta }$$

Where *λ* and *β* denote X-ray wavelength (0.15406 nm) and full width at half maximum (FWHM) respectively. Using equation (10), the micro-strain (*ε*) created in the thin films may be determined [25].

(10) $$\varepsilon =\frac{\beta \;\cot\;\theta }{4}$$

Where *θ* is Bragg's angle. Williamson and Smallman's relation as shown in equation (11) was used to calculate dislocation density for the non-isotropic distribution of thin films [26].

(11) $$\delta =\frac{n}{D_{hkl}^{2}}$$

Where *n* equals unity to give minimum dislocation density and *D*_hlk_ is the calculated crystallite size along the desired [hkl] direction. Using the appropriate formulae, the calculated structural parameters for both sputtered and annealed CZTS thin films, with various deposition pressures, are obtained. These results are arranged in Table 7.

**Table 7:** Particle size and RMS roughness of the CZTS thin films deposited at different deposition pressure.

Sample name	Particle Size (nm)	Roughness (nm)
C5	47	1.268
C10	57	1.461
C15	42	1.218
C20	61	2.339

Each crystal plane's complete reflection results in peaks at certain intensities in an XRD pattern. Therefore, it can be concluded from Fig. 4 that the peak intensity along the (112) direction first rises with deposition pressure (from C5 to C10) before declining again from C15 to C20 mTorr. CZTS thin film C10 has the highest peak intensity indicating the highest crystallinity. The crystallite size also reaches a maximum value within the range from C5 to C15 thin films. However, the crystallite size of the film C20 has increased. Fig. 4 shows that the peak intensity decreases from C10 to C20 mTorr, which confirms the reduction in crystallinity. Also, from Table 5 it can be observed that the stoichiometry was not met by the CZTS thin film C20. According to the data presented in Table 6, the C20 films may have larger crystallite size.

The total reflection from (112) crystal plan is much less as the stoichiometric ratio was not met [27] which is confirmed in Table 5.

**Fig. 5:**
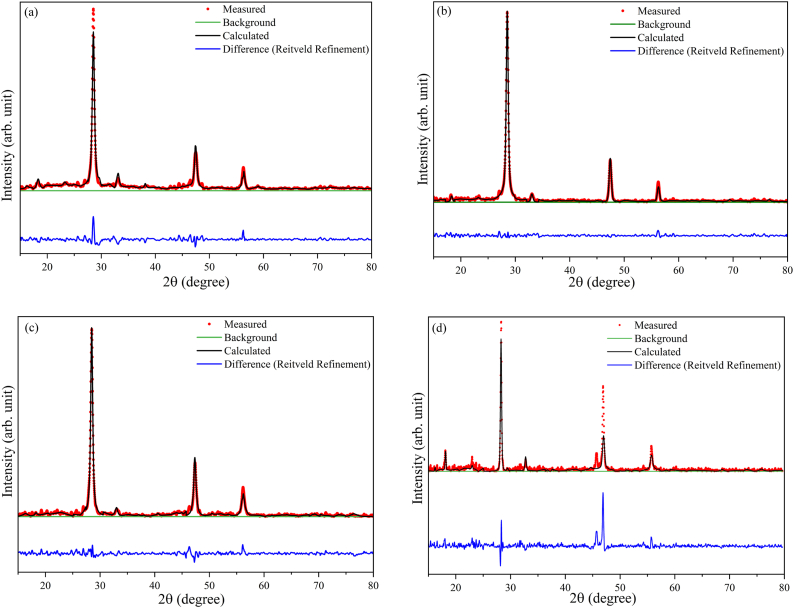
XRD data measured and Rietveld-refined graph of (a) C5 (b) C10 (c) C15 and (d) C20.

Fig. 5(a) through (d) shows the Rietveld refinement with acceptable fit quality (χ2 < 3) of the XRD patterns for C5 to C20 thin films respectively. Lattice parameters and crystallite size were obtained after refinement to estimate accurately and are listed in Table 6. The relation among the lattice parameters of a perfect tetragonal crystal structure is c = 2a [28]. Table 6 demonstrates that the lattice parameters a and c obtained from Rietveld refinement and the c/a ratio approaches a value of 2 for C10.

**Fig. 6:**
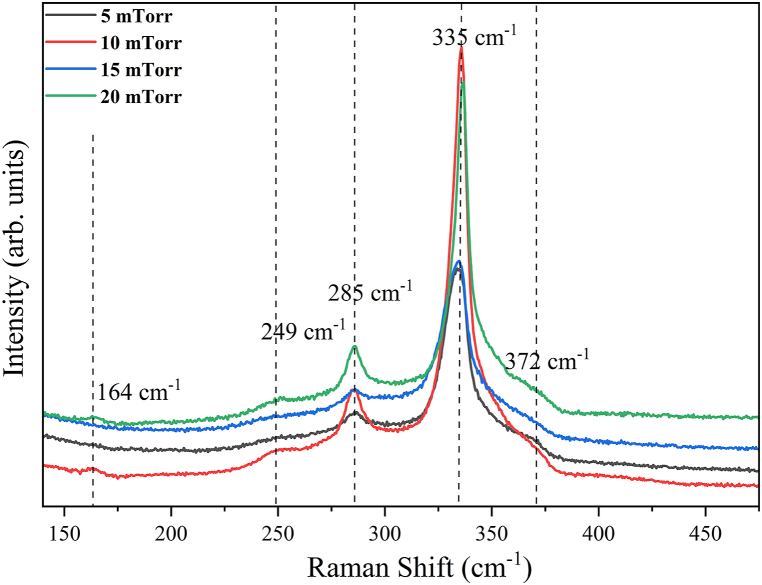
Raman spectra of C5 – C20 samples.

The investigation of the phase and composition of CZTS thin films after annealing was extended through the utilization of Raman scattering. This approach was necessary due to the challenge of distinguishing certain impurity peaks, which were obscured by the prominent CZTS peak, in the XRD pattern. Fig. 6 displays the Raman spectra of CZTS films fabricated using sputter deposition under different Ar pressures. In all of the annealed samples, three prominent peaks were detected at wavenumbers 285, 335, and 372 cm^−1^ that can be attributed to kesterite CZTS [29].

Additionally, it is clear that the major peak's intensity at 335 cm^− 1^ is the maximum for C10. In the Raman spectra of C10 and C20, distinct peaks were found at 164 and 249 cm^−1^. Peaks at 164 and 249 cm^− 1^ originated from cubic CuSnS_3_ and SnS_2_ secondary phases respectively [30,31], while the CZTS thin films deposited at 5 and 15 mTorr Ar pressure showed less existence of secondary phases. The occurrence of CuSnS_3_, SnS_2_, and SnS secondary phases in CZTS thin films can be attributed to the increased Sn concentration, as previously observed by Yang et al. [32].

### 3.4 Surface morphological analysis

**Fig. 7:**
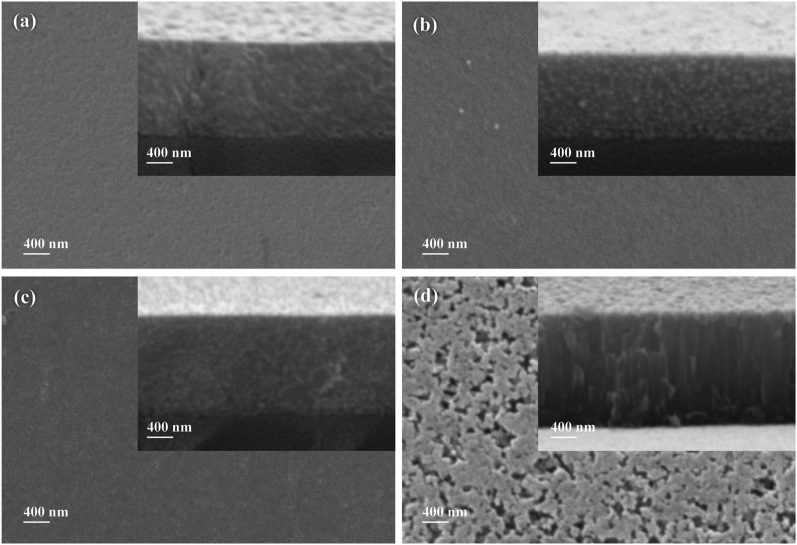
SEM images of CZTS thin films (surface and cross-sectional morphology) (a) C5 (b) C10 (c) C15 and (d) C20.

Fig. 7 presents the morphology of the top surface and cross-section of CZTS thin films sputtered at various Ar flows. The thickness of the films was adjusted to 850 nm. The cross-sectional morphology of CZTS thin films is compact and void-free.

The built-in electric field might be weakened by voids in the absorber layer, which eventually harms the device's functionality [32]. The cross-sectional images reveal that there is no existence of cracks in the fabricated thin films. Fig. 7 makes the influence of deposition pressure on surface morphology quite evident. C5, C10, C15 thin films, as shown in Fig. 7(a) through (c), have a uniform and pore-free surface overall. Whereas the surface of C20 is uniformly porous. Cross-sectional morphology also gives us a clear view of the effect of deposition pressure on the particle size. Large and spherical-shaped particles can be observed in C10 thin film compared to C5 and C15. Whereas the C5 and C15 have much smoother cross-sectional morphology with finer particle size. Fig. 7(d) confirms that C20 thin films have the largest particle size. These morphological observations completely agree with the XRD data analysis.

**Fig. 8:**
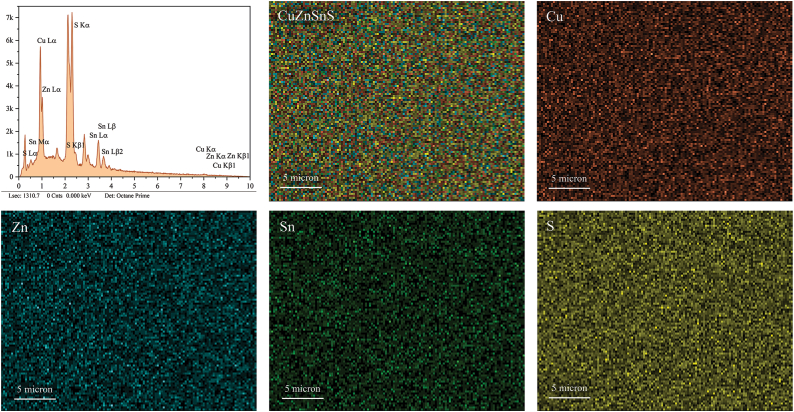
X-ray elemental cartography of C10 thin film: (a) EDS spectrum; (b) combined distribution of Cu, Zn, Sn, and S; (c) individual distribution of Cu; (d) individual distribution of Zn; (e) individual distribution of Sn; (f) individual distribution of S.

Furthermore, Fig. 8 presents the elemental cartography of the C10 thin film. In the cartography images, each element- Cu, Zn, Sn, and S- is assigned a distinct color (red, blue, green, and yellow, respectively) in the individual elemental maps shown in Fig. 8(c) through 8(f). The combined distribution of these elements is depicted in Fig. 8(b), while Fig. 8(a) shows the EDS spectrum. The high homogeneity in the distribution of Cu, Zn, Sn, and S across the surface of the CZTS thin film is clearly observed, indicating a well-mixed and uniform composition.

**Fig. 9:**
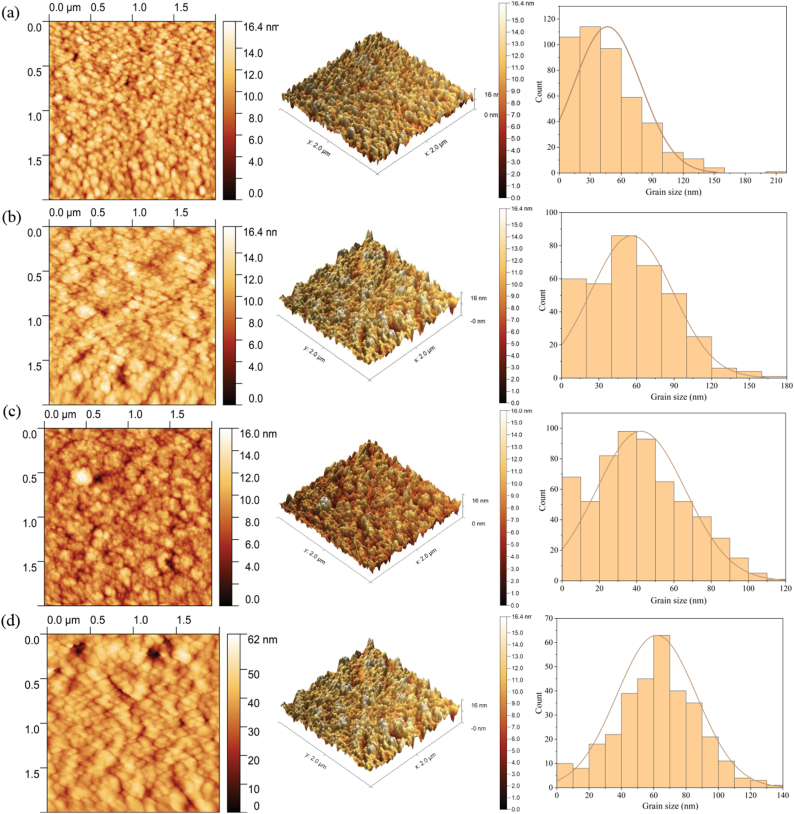
Two-dimensional, Three-dimensional AFM image with the histogram of the particle size distribution of CZTS thin films (a) C5 (b) C10 (c) C15 and (d) C20.

In the AFM images of Fig. 9 the impact of deposition pressure on CZTS grain development is visible. Fig. 9(a) through 9(d) show the surface morphology and particle size distribution of the films deposited at different pressures: C5, C10, C15, and C20, respectively. The particle sizes of the CZTS thin films are 47, 57, 42, and 61 nm for the C5, C10, C15, and C20 thin films, respectively. These findings concur with the XRD data (Table 6) and SEM images (Fig. 7). The crystallite size distribution of CZTS thin films, as demonstrated in histogram images, indicates that the films have a monodispersed grain distribution. AFM image analysis for the C5-C20 thin films is summarized in Table 7. It is observed that RMS roughness is related to the particle size of the CZTS thin films [33]. The grain boundary shrinks as the grain size grows, increasing the minority charge carrier's effective diffusion length and short-circuit current. Larger grains are advantageous because the size of the absorber layer's grains may rise the performance of solar cells. According to Table 7, C5 and C15 films have the smoothest surfaces and have little grains and low RMS roughness values. C10 thin films have larger grain size with larger value of RMS roughness compared to C5 and C15. Chaudhari et al. also observed similar results in their study [33]. Whereas the SEM images in Fig. 7 demonstrate the porous morphology which agrees with AFM image analysis resulting the largest RMS value for C20.

### 3.5 Optical properties

Equation (12) was used to determine the optical absorption coefficient (α) from transmittance and spectra [34].

(12) $$\alpha =\frac{1}{t}\ln\;\left[\frac{{\left(1-R\right)}^{2}}{T}\right]$$

where *T, R* and *t* refer to transmittance, reflectance and thickness respectively.

**Fig. 10:**
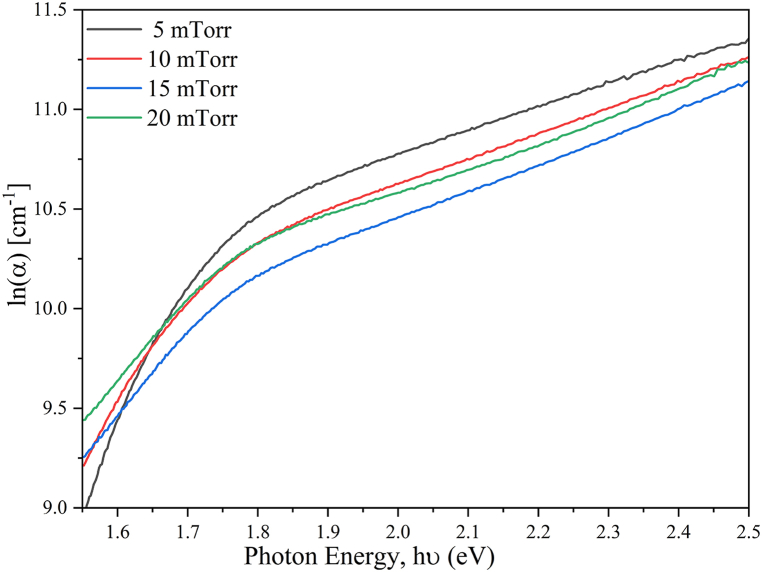
Natural logarithm of optical absorption coefficient (α) as a function of photon energy (hν) of the CZTS films by sputtering at different deposition pressure.

The optical absorption coefficient (α) of the CZTS thin films is shown in Fig. 10.

The visible region's absorption coefficient is greater than 10^4^ cm^−1^, which is consistent with data from previous publications [35] and consequently, it is assumed that the film can be a suitable component for photovoltaic solar energy conversion. The energy band gap is deduced by extrapolating the linear (αhν)^2^ versus hν plots to (αhν)^2^ = 0, as depicted in Fig. 10**.**

Plotting *(αhν)*^*2*^ with respect to incoming photon energy will enable the Tauc relation as depicted in equation (13), to be used to determine the direct optical energy bandgap (E_g_) [24].

(13) $${\left(\alpha h\nu \right)}^{2}=\left(h\nu -E_{g}\right)$$

**Fig. 11:**
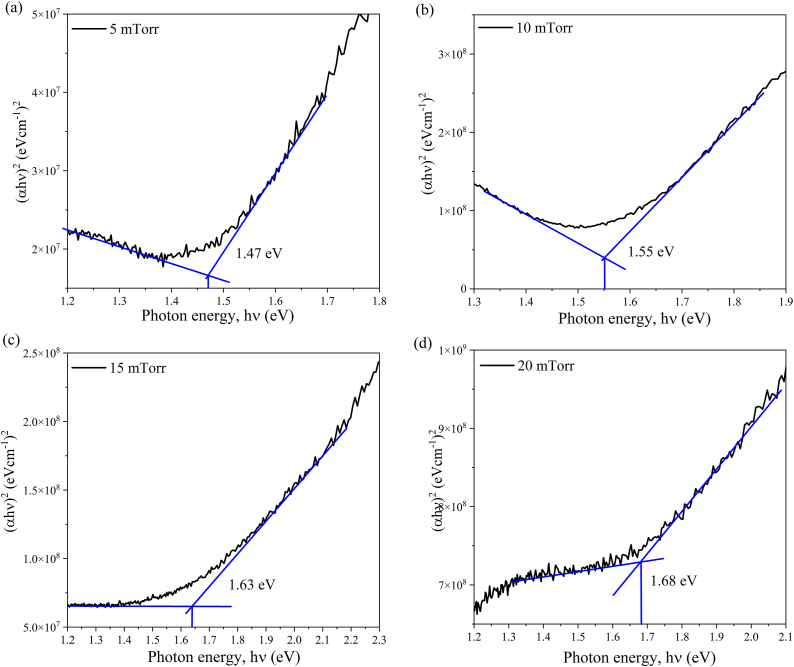
Band gap of the CZTS thin films (a) C5 (b) C10 (c) C15 and (d) C20.

To get the optical energy bandgap of each film, the linear part of the curve is projected to *(αhν)*^*2*^*=0* in Fig. 11.

Fig. 11 shows the band gap of CZTS thin films (a) C5, (b) C10, (c) C15, and (d) C20, demonstrating the variation in band gap values with deposition pressure. As observed, the band gap of the CZTS thin films increases from 1.47 eV to 1.68 eV with increasing deposition pressure. A linear fit utilized as an abscissa is employed for the slope beneath the fundamental absorption. The junction of the two fitted lines provides an assessment of the band gap energy [36]. The baseline trend assists in distinguishing the authentic onset of absorption, resulting from electrical transitions across the bandgap, from any low-energy background absorption, such as defect states, impurity levels, or noise that does not directly indicate the bandgap. Consequently, the baseline trend offers a definite reference for extrapolation, thereby enhancing the precision of the expected bandgap value [36]. The broader band gap seen in the CZTS thin films is likely attributable to the structural and compositional alterations inherent to the thin films. Factors like as non-stoichiometry and defects, including vacancies or interstitials, generate localized states that alter the band edges and similar results were reported in Refs. [27,34,37]. Furthermore, secondary phases may modify the electrical characteristics of the film. The band gap of the thin films may be influenced by the stoichiometry and the presence of secondary phases. The XRD pattern depicted in Fig. 4 confirms the existence of SnS phase. The increase in peak intensity with deposition pressure signifies enhanced incorporation of the SnS phase, perhaps leading to a progressive rise in the band gap, as documented in Ref. [38]. Fig. 6 illustrates that the Raman spectra exhibit a consistent trend, wherein the relative intensity of the CuSnS_3_ and SnS_2_ secondary phases escalates with increasing deposition pressure. Table 5 indicates that the Zn/Sn ratio similarly increases with deposition pressure. Zinc atoms are smaller than tin atoms. Consequently, the bond lengths inside the crystal lattice may change when Zn substitutes Sn. Reduced bond lengths attributable to Zn's decreased atomic radius may yield stronger bonds. Enhanced bonding may thus elevate the energy disparity between the valence band and conduction band, so broadening the band gap. CZTS thin films deposited at 10 mTorr chamber pressure have an optical bandgap of around 1.55 eV. This value is quite near to the single-junction solar cell's theoretically ideal value. The aforementioned optical properties suggest that C10 can be a dependable option for highly efficient absorber layer.

### 3.6 Electrical properties

**Fig. 12:**
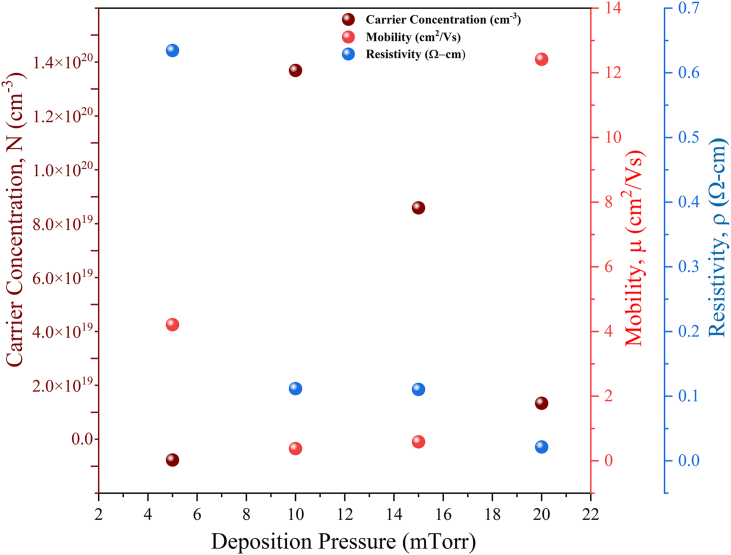
Carrier concentration, mobility, and resistivity of the C5 to C20 CZTS thin films.

The CZTS thin films carrier concentration, mobility, and resistivity are displayed in Fig. 12. The fact that all of the carrier concentration readings were positive indicates that the fabricated films had p-type conductivity. When the films are deposited at increased pressure, the Hall mobility (μ) is decreased.

Given that the size of the crystallites is anticipated to be smaller at a high working pressure during deposition, the reduced mobility may be caused by an increase in grain boundary scattering [22]. This would occur because the sputtered neutrals have less kinetic energy when they reach the substrate due to enhanced dispersion and hence have less energy to move over the substrate surface. The mobility was found from 0.3806 to 12.42 cm^2^/Vs. Fig. 12 depicts that there is a decrease in mobility from 5 to 15 mTorr. However, the mobility increase for C20 may be the result of a growth in crystallite size, which is supported by XRD data. A larger crystallite size also results in a minimum resistivity value for the CZTS thin films deposited at 20 mTorr. With an increase in deposition pressure, the resistivity has demonstrated a tendency toward diminishing. The XRD data can be correlated with this decrease in resistivity. The literature makes it clear that a bigger crystallite size can result in a reduction in the films' resistivity [39].

The value of resistivity varies from 0.6344 to 0.02153 Ω-cm. The layer deposited at a pressure of 5 mTorr Ar deposition pressure showed the highest resistivity. A maximum carrier concentration was measured for C10. The sputtered species have numerous collisions at high pressure, which causes the film-forming particles to heat up. As a result, the ions energy decreases and the bonding energy weakens, which has a negative impact on the films crystallinity. But if the pressure is too low, it is challenging to discharge the system and the plasma becomes unstable [22]. XRD and Raman patterns confirm the good crystallinity of C10. Each bulk carrier density varies from $2.34\times 10^{18}$ to $1.47\times 10^{20}$ N(cm^−3^), complying with early reported values [38].

## 4 Conclusions

In conclusion, the co-sputtering deposition method employed in this study for the fabrication of Cu_2_ZnSnS_4_ (CZTS) thin films using copper (II) sulfide (CuS), zinc sulfide (ZnS), and tin sulfide (SnS) targets on a soda lime glass substrate has yielded valuable insights. The careful selection of targets guarantees the presence of an abundant quantity of volatile S material in the as-deposited CZTS thin films. The subsequent annealing process at 470 °C in the absence of a specific sulfur source has led to noteworthy outcomes. When the deposition pressure is increased to 5–20 mTorr, there is a tendency toward larger crystallite sizes. For the film deposited at 20 mTorr, the greatest crystallite size is observed to be 437 Å, with a lattice parameter of a = 5.471 and c = 10.931. Furthermore, for the same sample, SEM investigation shows the maximum particle size of 61 nm with a greater roughness of 2.399 nm. The band gap, which ranges from 1.43 to 1.68 eV, likewise exhibits a rising trend with pressure. On the other hand, resistivity exhibits diminishing tendencies against rising pressure within the range of 0.6344 to 0.0215 Ω-cm. At 5 mTorr Ar deposition pressure, the film with the highest resistance developed. The sample obtained at 10 mTorr exhibited a peak carrier concentration of approximately 1.47 × 10^20 N/cm³. The band gap (1.55 eV) and stoichiometric atomic ratio (2.1:1:1:4) approximate the conventional values for the same sample. Effective stoichiometry guarantees balanced lattice stability and optimum band alignment, resulting in low recombination loss. The band gap value enables the absorption of a substantial section of the solar spectrum, rendering the produced thin films well optimized for photovoltaic systems. These cause-effect correlations, elucidated through comprehensive experimental data, contribute significantly to the understanding of the deposition process and the resulting film properties. Researchers and practitioners in this field are likely to find substantial interest in the nuanced findings presented here, paving the way for further exploration and optimization of CZTS thin films for potential applications in photovoltaic and optoelectronic devices. In essence, this study opens avenues for exploring CZTS thin film fabrication methods that deviate from conventional sulfurization approaches to unlock the full potential of this simplified fabrication technique.

## CRediT authorship contribution statement

**Munira Sultana:** Writing – original draft, Visualization, Investigation, Formal analysis, Data curation. **Afrina Sharmin:** Writing – review & editing, Visualization, Investigation. **Md Rashed Alam:** Investigation, Formal analysis. **Shahran Ahmed:** Investigation, Conceptualization. **Md Aftab Ali Shaikh:** Writing – review & editing, Funding acquisition. **M.S. Bashar:** Validation, Supervision, Resources, Methodology, Investigation, Conceptualization.

## Data availability statement

The dataset generated for analysis during the current study is available from the corresponding author on reasonable request.

## Declaration of competing interest

The authors declare that they have no known competing financial interests or personal relationships that could have appeared to influence the work reported in this paper.
